# Non-ocular biomarkers for early diagnosis of diabetic retinopathy by non-invasive methods

**DOI:** 10.3389/fendo.2025.1496851

**Published:** 2025-03-12

**Authors:** Huaixue Gou, Juping Liu

**Affiliations:** Tianjin Key Laboratory of Retinal Functions and Diseases, Tianjin Branch of National Clinical Research Center for Ocular Disease, Eye Institute and School of Optometry, Tianjin Medical University Eye Hospital, Tianjin, China

**Keywords:** diabetic retinopathy, biomarkers, non-ocular, non-invasive, community screening, nailfold capillaroscopy, skin autofluorescence

## Abstract

Diabetic retinopathy (DR) is the predominant vision-threatening complication in individuals with diabetes mellitus. Timely diagnosis and intervention facilitate the prevention of diabetes-associated visual impairment. Classical imaging methods may prevent the timely detection of DR due to shortages of specialized facilities and retinal specialists, particularly in remote areas. In recent years, research on biomarkers related to DR has rapidly developed, playing an important role in risk assessment and early detection of the disease. Some ocular biomarkers from the vitreous body or aqueous humor were invasive, which hampered their application in clinical practice. Meanwhile, biomarkers based on omics were limited by their uneasily accessible use and complicated variables with a relatively low degree of reproducibility. As modern technology progresses, advanced non-ocular biomarkers of DR have established a comprehensive platform for the prompt identification of DR, independent of ophthalmic professionals or devices and accessible to non-ophthalmologists during community screenings. This review focuses on biomarkers derived from non-ocular sample sources, such as nailfold and skin, accessible through non-invasive methods, to reveal if they can be considered as an effective option for the early identification of DR by non-ophthalmologists in community screening initiatives.

## 1 Introduction

Diabetic retinopathy (DR) is a retinal microangiopathy resulting from the chronic effects of diabetes mellitus (DM), which is the predominant sight-threatening complication in patients with DM (1, 2). Recent estimates indicated that the global prevalence of DR among adults with diabetes was 22.3%, with 6.2% of these patients exhibiting vision-threatening diabetic retinopathy (VTDR) (3). In addition to its visual impacts that may result in a lower quality of life, DR has been associated with an elevated risk of systemic vascular problems (4), hence adding a significant burden on individuals and healthcare systems (5). DR can be divided into non-proliferative DR (NPDR) and proliferative DR (PDR). In the initial stage of the disease, it mostly presents as NPDR with mild vascular hyperpermeability. As the disease progresses, NPDR develops from mild to severe with retinal capillary leakage or loss, causing retinal ischemia. Patients with NPDR are typically asymptomatic, making it difficult to detect the disease at an early stage. When the condition further deteriorates, it will progress to the PDR stage with new vessel growth on the optic disc and retina, when the patient may present with a sudden loss of vision due to a vitreous hemorrhage (6). In addition, diabetic macular edema (DME) also plays an important role in the development of DR. DME refers to the retinal thickening in the posterior pole and may occur in either NPDR or PDR. When patients develop DME, their vision gradually declines, severely affecting their quality of life. Consequently, early detection of DR is essential for reasonable risk categorization, early management, optimizing therapeutic effects, and lowering healthcare expenditures.

The prevailing standard of treatment is for an annual dilated retinal examination to facilitate earlier diagnosis (7, 8). The detection of DR mostly employs conventional procedures such as direct or indirect ophthalmoscopy, fundus photography, or optical coherence tomography (OCT). Still, these techniques are protracted and manual and require specialized equipment and retinal experts, leading to resource deficiencies, especially in screening capacity and retinal specialist availability, which may hinder the early identification of DR.

**Figure 1:**
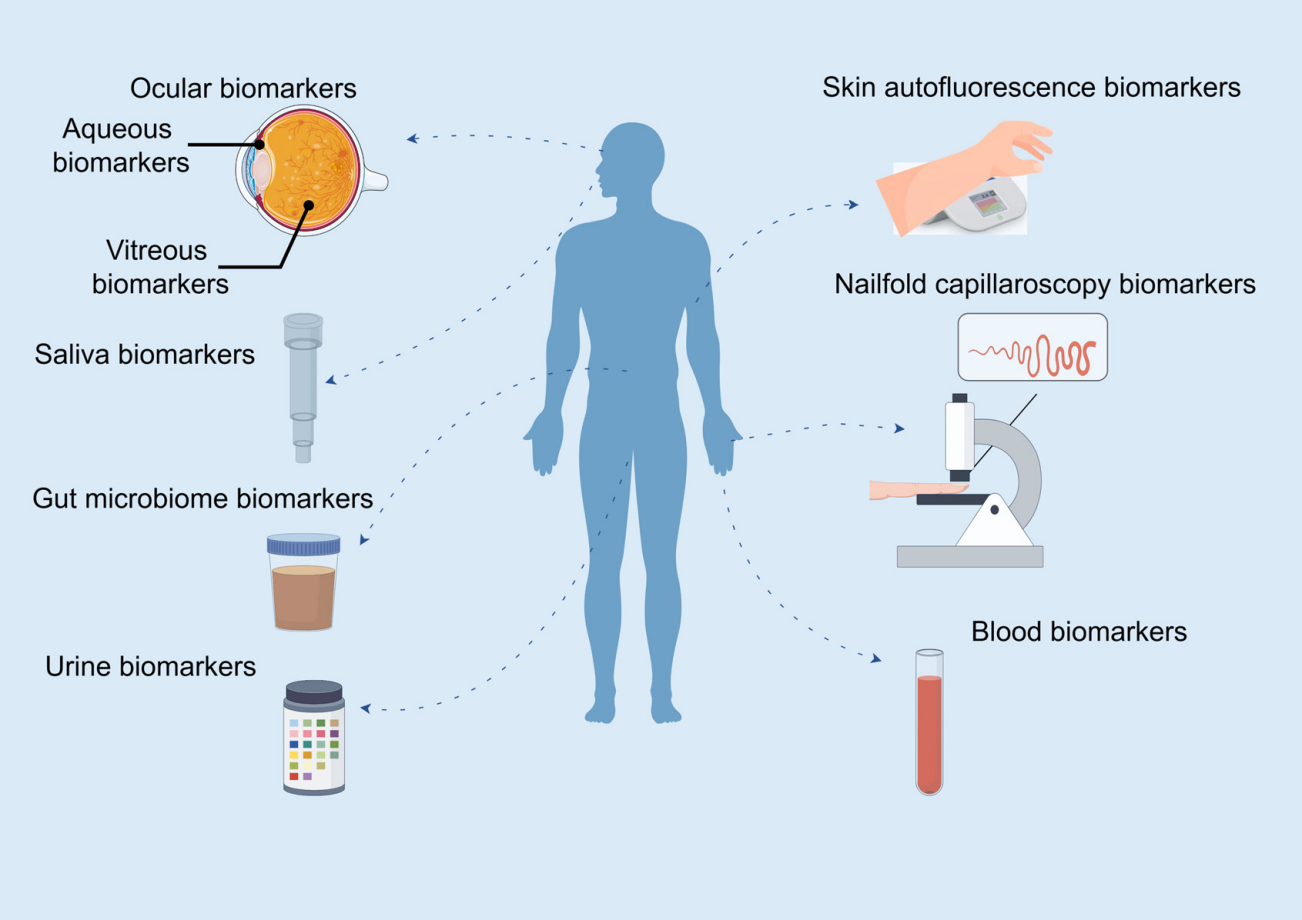
Biomarkers from various sample sources in diabetic retinopathy. By Figdraw.

Recently, multiple unique biomarkers from various sample sources connected with DR were discovered, which was essential for early identifying the occurrence and development of DR (**Figure 1**). Some ocular biomarkers from the vitreous body or aqueous humor (9) are invasive and thus undesirable for both patients and clinicians. In recent years, the rapid advancement of translational medicine has given rise to the investigation of biomarkers from non-ocular sample sources, including nailfold, skin, blood (10–13), saliva (14), feces (15), and urine (16), for the clinical diagnosis and prognosis of DR. These biomarkers are both safe and readily accessible for sample collection, particularly in difficult groups. Non-coding RNAs (17) and genomic (18) and lipidomic (19) biomarkers have also been intensively investigated to predict the risk of DR development, but most of them are limited by their uneasily accessible use and complicated variables with a relatively low degree of reproducibility, preventing their use in clinical practice, particularly in community hospitals or remote areas where ophthalmic investigations are not readily available. Given the foregoing, there is a need for easy, rapid, reliable, and affordable non-ocular biomarkers in community-based DR screening that does not require ophthalmic specialists or ophthalmic technology and may be performed by non-ophthalmologists.

To our knowledge, there is a paucity of studies that have comprehensively assessed non-ocular diagnostic biomarkers of DR and their application in clinical practice. Optimal biomarkers are characterized by specificity and can be readily assessed by non-invasive techniques (20). Therefore, this review concentrates on non-ocular biomarkers, easily obtained through non-invasive methods, to explore their potential as an effective option for non-ophthalmologists to identify DR early in community screening initiatives.

## 2 Nailfold capillary changes

Nailfold capillaroscopy (NFC) is an innovative non-invasive method for detecting systemic disease-related microvascular morphological and functional abnormalities (21). Some studies have established the relationships between nailfold capillary changes and the existence and severity of DR. Changes such as tortuosity (curvature of the capillary limb without crossover), avascular regions (absence of two or more adjacent capillaries in the most distal row), branching capillary (numerous small buds emerging from the distal loop), diminished capillary density, and microhemorrhages were markedly elevated in individuals suffering from DR compared to those DM without DR (22–25). Furthermore, Shikama et al. (26) revealed that individuals with T2DM had an elevated risk of DR correlated with an increased number of crossing capillaries. When data on crossing capillaries were added to a model containing known risk and inhibiting factors for DR (age, sex, diabetes duration, glycated hemoglobin, systolic blood pressure, body mass index, and use of certain medications), the Nagelkerke *R* ^2^ increased from 0.29 to 0.35. As the value of Nagelkerke *R* ^2^ ranges from 0 to 1, the closer it is to 1, the better the model fits the data, suggesting that crossing capillaries might serve as a novel biomarker related to the residual risk of DR.

Subgroup studies of DR patients indicated that alterations in nailfold capillaries were substantially correlated with the degree of DR and may run parallel to retinal changes. Several cross-sectional studies found that abnormalities in nailfold capillaries, including diminished capillary density, tortuosity, a larger number of mega capillaries, and dilated apical capillaries, were observed in patients with PDR, significantly greater than in patients with NPDR and those without DR (24, 25, 27). These findings implied that NFC can detect the microvascular changes in the nailfold capillaries accurately, which were predominantly proliferative in the early stages and regressive in the advanced stages (23). Furthermore, previous studies identified that patients with DR who have a prolonged illness duration over 20 years or poor glycemic control (HbA1c > 11%) had significantly higher frequencies of mega capillaries, enhanced tortuosity, and neovascularization in type 2 diabetes mellitus (T2DM) (22, 24), which was also observed in research on juveniles with type 1 diabetes mellitus (T1DM) (28). It indicated that variations in nailfold capillaries may play a role in prognosis alone.

Upon diagnostic test estimation for DR detection, according to the data from a prospective study, Uyar et al. (22) used semiquantitative capillary assessments to evaluate the presence of tortuosity. The receiver operating characteristic (ROC) curve analysis of the diagnostic test estimation showed that the area under the curve (AUC) value for tortuosity was 0.615 (95% CI 0.540 to 0.689). For significance, in the multivariate logistic regression analysis, tortuosity was significantly associated with DR (OR 2.16; *P* = 0.036). In general, an AUC value ranges from 0 to 1. The closer to 1 indicates a better diagnostic performance, while a value of 0.5 means the test is no better than random chance. Here, an AUC of 0.615 for tortuosity suggests that it has limited diagnostic accuracy for detecting DR, despite the significance in the regression analysis. Although previous research offered new perspectives on the relationships between NFC changes and DR, it should be noted that prior studies were qualitative assessments, which were subjective and lacked the high-resolution quantification ability of the quantitative approach, giving rise to a lack of reliability in their outcomes.

Unlike previous research, Okabe et al. (29) evaluated nailfold capillary alterations quantitatively with NFC, Kekkan-Bijin SC-10, showing an AUC value for nailfold capillary length of 0.83 (95% CI 0.71 to 0.90, *P* < 0.001). Furthermore, when adding nailfold capillary length into conventional systemic risk factors, it also substantially enhances the discriminating capability for DR, yielding an AUC of 0.89 (95% CI 0.79 to 0.95, *P* < 0.001). Another study applied a video-based technique for nailfold capillary evaluation, revealing that vasodilation of the nailfold capillaries serves as a reliable indication of DR, reaching an AUC of 0.75 (95% CI 0.634 to 0.875, *P* < 0.001) (27). Similarly, in the study of Rohit et al. (23), the aforementioned characteristics were recorded by a semiquantitative NFC score in the center 3 mm of each image. They found that NFC score was an important predictor of DR with an AUC of 0.745 (95% CI 0.648 to 0.827, *P* < 0.001) for correctly predicting DR. Surprisingly, a high diagnostic accuracy of NFC (72%) in retinopathy was observed even in patients with controlled HbA1c levels (<7%) (23). Subsequently, for practical use in a clinical environment, Goydin et al. (30) created a computer program calculating the results of DR predictions according to NFC changes, showing that NFC changes (capillary network density, velocity of arterial and venous blood flow) have a high diagnostic information value for detecting both NPDR and PDR (92.2% vs. 94.4%). Automatic assessment of NFC changes eliminated the effects of grader subjectivity, making it possible for NFC changes to serve as essential non-invasive markers for DR identification.

Overall, the quantitative evaluation of NFC has offered a variety of metrics for the direct assessment of peripheral microvascular structure and has the potential to serve as a promising supplementary method for DR identification due to its high sensitivity and specificity, especially when added to conventional systemic risk factors. To some degree, NFC also seems to have a role in the prognosis and identification of patients at elevated potential for DR. In addition, through a monitor, patients can confirm their microvascular damage visually, which may enhance diabetes self-management and compliance with DR screening.

Despite these favorable findings, current studies still had the following limitations that require further consideration. First, the existing research mainly consists of observational studies, making it difficult to analyze the temporal relationship between NFC alterations and DR. Additionally, due to the relatively small sample size of these studies, it can be difficult to apply these present results to all geographic regions and ethnic groups. As a result, larger cohort studies with diverse demographic populations are required to elucidate the precise and temporal relationships between NFC changes and DR. Simultaneously, alterations in NFC could indicate multiple diseases, potentially resulting in an erroneous diagnosis. Future NFC research is required to elucidate the link between various comorbidities and DM.

## 3 Skin autofluorescence changes

Previous research *in vitro* has verified that advanced glycation end-products (AGEs) play a crucial part in DR pathogenesis by inducing pericyte apoptosis (31), increasing proinflammatory mediators (32), hindering retinal microvascular endothelial cell function (33), and adding VEGF secretion (34), thereby facilitating the development of DR. Consequently, AGEs have the potential to function as a biomarker for DR.

Given the low turnover rate, AGEs frequently accumulate in skin tissues over time. The direct measurement of AGEs in skin biopsy specimens can be a marker of future DR development in the DCCT/EDIC study (35). While the direct evaluation of AGE quantification from certain targeted tissues has enhanced our understanding of the correlation between AGE accumulation and the degree of DR, the fact that this method is invasive and time-consuming still impedes its clinical application.

With the unique fluorescence pattern of AGEs, skin autofluorescence (SAF), a non-invasive biomarker for AGE accumulation in epidermal tissues, has recently been developed (36). In recent years, AGEs quantified via SAF have been employed for medical diagnosis (37, 38), particularly in individuals with diabetes (39). Studies to date have suggested that SAF is a non-invasive surrogate biomarker for diabetic microvascular complications (40, 41), as it provides a non-invasive and cost-effective diagnostic method with a high level of reproducibility (37).

Most earlier studies on the correlation between DR prevalence and SAF have focused on T1DM patients. It has been established that SAF correlates with the prevalence of DR in adults (42, 43), as well as in adolescents with T1DM (44). Skin AGE accumulation was also predictive of future DR progression (35). The high area under the ROC curve for DR (AUC: 0.89, 95% CI 0.85 to 0.94) observed by Januszewski et al. (51) suggested that SAF may function as a non-invasive biomarker for DR in T1DM.

Consistent with previous research on T1DM patients, in T2DM patients, SAF was also correlated with the severity of DR (45, 46). After adjusting for variables such as age, smoking, and diabetic nephropathy, which were known to increase SAF measures (42), SAF remained linked with the severity of DR in T2DM patients (47). Also, a current meta-analysis of six studies revealed that SAF was connected to DR and that for every 0.1 unit rise in SAF level, there was a 5% increased risk of DR (OR = 1.05, 95% CI 1.03 to 1.08) (41). Differently, it is unclear whether there is a linear correlation between the severity of the DR in T2DM patients and the degree of AGEs. Takayanagi et al. (48) evaluated the potential role of AGEs during the progression of DR in T2DM patients and discovered that among groups categorized by quartiles of AGE scores, only the top quartile exhibited a substantially elevated rate of PDR. This finding suggests that significantly raised levels of AGEs may contribute to the development of PDR, highlighting the clinical value of the SAF as a non-invasive and dependable biomarker for individuals at risk of VTDR.

Most importantly, accumulating evidence in recent years suggested that SAF was a superior diagnostic biomarker to HbA1c for DR and PDR. Concretely, an ROC analysis revealed that the predictive capacities of SAF and HbA1c for DR were 0.79 and 0.55, while for PDR, the predictive capacities were 0.81 and 0.61, respectively (45). Furthermore, Ling et al. (46) found that sensitivity was considerably greater than that of HbA1c when AGEs >72.3. AGEs demonstrated a considerably greater efficacy in early diagnosis than HbA1c in the context of VTDR. These might be because of the “metabolic memory” effect, where the accumulation of AGEs shows the long-lasting consequences of hyperglycemia, while HbA1c reflects the short-term effects of glycemic management. Given that microvascular issues can arise even in prediabetes, SAF could therefore be more useful than HbA1c for assessing retinal damage (35).

As for the accuracy of SAF for DR detection, the SAF test as a diagnostic tool for DR has demonstrated adequate accuracy for clinical application. However, the ideal cutoff value of SAF for distinguishing patients remains controversial. In a study including 138 T2DM patients, Hirano et al. (47) revealed that the cutoff point between mild NPDR and no DR was 2.25 with an AUC of 0.78, while the cutoff line between severe NPDR and PDR was 2.32 (AUC: 0.75). Another study of 1,471 T2DM patients found that the best cutoff point for SAF to identify any DR was 72.3 (AUC: 0.56) and VTDR was 77.1 (AUC: 0.73) (46). A recent meta-analysis encompassed four studies to assess the efficacy of SAF as an instrument for DR screening. The OR value for detecting DR with SAF was 5.11 (49). These discrepancies may be due to different sample sizes, subject variability, and lifestyle variances. Notably, larger samples and further studies are required to determine suitable SAF reference values across populations.

Considering the widespread agreement that SAF is a potential biomarker for the onset and development of DR, multiple confounding variables should be evaluated before they are used in clinical trials. Firstly, in individuals with T2DM, renal function could affect the correlation between SAF and retinopathy, as renal insufficiency alone can elevate SAF levels even in non-diabetic individuals (50, 51). Consequently, in reality, SAF may assist in identifying certain participants for DR screening; however, individuals with renal insufficiency and diabetes should be examined regardless of their SAF levels, as they exhibit a three-fold greater prevalence of retinopathies (52), not associated with SAF (53). Furthermore, SAF can be impacted by skin pigmentation, resulting in diminished measurement accuracy (54). Fortunately, the AGE sensor may make up for this deficiency (55). Subsequently, Kim et al. (56) tested a novel SAF measuring system transmitted through the first dorsal interossei muscles, which provided better screening performance, especially in Asian T2DM subjects. When measuring SAF, it is essential to consider other confounding factors, such as the heightened generation or intake of AGEs from dietary sources or smoking, which could exacerbate AGE buildup, alongside the diminished elimination of AGEs (57–59).

Generally, the SAF measurement of AGEs as a diagnostic biomarker for DR is accurate enough for clinical use; however, it cannot replace the fundus test due to its limitations. In order to address its variability and ensure its appropriate use, more patient evaluations are required. Additionally, we also need more research to establish reference cutoffs and account for all the variables that could influence the test.

## 4 Discussion and conclusion

In general, NFC changes and SAF are sensitive, cost-effective biomarkers that enable the early detection of DR **Table 1**. Quantitative evaluation of NFC, such as the nailfold capillary length and arterial blood flow velocity, showed distinct advantages in differentiating the different stages of DR. Furthermore, adding these biomarkers to risk factors can enhance the discrimination of DR. Moreover, in both T1DM and T2DM patients, SAF has been shown to be correlated with the prevalence and severity of DR. Furthermore, SAF may be a superior diagnostic biomarker to HbA1c for DR and PDR, as it reflects long-term hyperglycemia effects. As both NFC changes and SAF are sensitive and cost-effective biomarkers for early DR detection, more longitudinal studies and basic research are needed to clarify their relationships with DR, validate them in independent cohorts, and establish appropriate reference values.

**Table 1:** Summary of key articles about non-ocular diagnostic biomarkers of DR by non-invasive methods.

Author and date	Study type	Sample type and size	Biomarker	Diagnostic outcome	References
Nailfold capillary changes					
Uyar S et al., 2016	Prospective study	✓216 patients with T2DM (93 with DR, among which 62 had PDR and 31 had NPDR; 123 without DR) ✓101 healthy controls	✓Tortuosity (assessed by NFC)	For DR ✓Tortuosity: AUC was 0.615 (95% CI 0.540–0.689)	(22)
Abd-El-Khalik DM et al., 2022	Cross-sectional study	✓62 patients with T2DM (26 with DR, among which 4 had PDR and 22 had NPDR; 36 without DR)	✓Capillary width (assessed by nailfold video capillaroscopy)	For DR ✓Capillary width: AUC was 0.754 (95% CI 0.634–0.875, *P* < 0.001) ✓The cutoff value was 27.8 µm (sensitivity 42.3%; specificity 94.4%)	(27)
Raina R et al., 2023	Cross-sectional study	✓100 patients with T2DM (54 with DR and 46 without DR)	✓Total NFC score (assessed by a handheld dermatoscope)	For DR ✓Total NFC score: AUC was 0.745 (95% CI 0.648–0.827, *P* < 0.001) ✓The cutoff value was > 0 (sensitivity 51.85%; specificity 95.65%)	(23)
Okabe T et al., 2023	Cross-sectional study	✓83 patients with T2DM (93 with DR, among which 29 had PDR and 24 had NPDR; 30 without DR) ✓63 healthy controls	✓NC number ✓NC length ✓NC width ✓Turbidity (assessed by the Kekkan-Bijin SC-10 device and Capillary Analysis System program)	For DR ✓NC number: AUC was 0.66 (95% CI 0.53–0.77, *P* = 0.03); ✓NC length: AUC was 0.83 (95% CI 0.71–0.90, *P* < 0.001); ✓NC width: AUC was 0.79 (95% CI 0.67–0.87, *P* < 0.001); ✓Turbidity: AUC was 0.78 (95% CI 0.66–0.87) For PDR ✓NC number: AUC was 0.70 (95% CI 0.55–0.82, *P* = 0.004); ✓NC length: AUC was 0.83 (95% CI 0.70-0.91, *P* < 0.001); ✓NC width: AUC was 0.80 (95% CI 0.66–0.89, *P* < 0.001); ✓Turbidity: AUC was 0.76 (95% CI 0.62–0.86, *P* < 0.001)	(29)
Goydin AP et al., 2023	Cross-sectional study	✓90 patients with T2DM (60 with DR, among which 29 had PDR and 31 had NPDR; 30 without DR)	✓Arterial blood flow velocity ✓Venous blood flow velocity ✓Capillary network density (assessed by a computerized capillaroscope KK-01)	For NPDR ✓Arterial blood flow velocity: AUC was 0.994, *P* < 0.0001 ✓Venous blood flow velocity: AUC was 0.982, *P* < 0.0001 ✓Capillary network density: AUC was 0.846, *P* < 0.0001 For PDR ✓Arterial blood flow velocity: AUC was 0.941 ✓Venous blood flow velocity: AUC was 0.909 ✓Capillary network density: AUC was 0.963	(30)
Skin autofluorescence changes					
Tanaka K et al., 2020	Cross-sectional study	✓269 patients with T1DM ✓114 healthy controls	SAF measured with an AGE reader from unscarred skin on the volar surface of each arm, corrected for skin color, and the mean of six readings in arbitrary units (AU)	For DR ✓SAF: AUC was 0.89 (95% CI 0.85–0.94)	(51)
Yasuda M et al., 2014	Case–control study	✓67 patients with T2DM (52 with DR, among which 21 had PDR and 31 had NPDR; 15 without DR) ✓67 healthy controls	SAF measured with an AGE reader on the volar side of the lower right arm, expressed in arbitrary units (AU)	For DR ✓SAF: AUC was 0.79 (95% CI 0.61–0.91) For PDR ✓SAF: AUC was 0.81 (95% CI 0.70–0.92, *P* = 0.029)	(45)
Ying L et al., 2021	Cross-sectional study	✓1471patients with T2DM (372 with DR, among which 332 had mild to moderate NPDR; 4 had severe NPDR and 36 had VTDR; 1,099 without DR)	SAF measured with an AGE reader	For DR ✓SAF: AUC was 0.560 (95% CI 0.534–0.586) For mild to moderate NPDR ✓SAF: AUC was 0.530 (95% CI 0.505–0.556) For VTDR ✓SAF: AUC was 0.728 (95% CI 0.704–0.750)	(46)
Hirano T et al., 2014	Cross-sectional study	✓138 patients with T2DM (102 with DR, among which 31 had PDR and 71 had NPDR; 36 without DR) ✓111 healthy controls	SAF measured with an AGE reader on the ventral side of the lower arm	For predicting the difference between mild and moderate NPDR ✓SAF: AUC was 0.78 with a sensitivity of 80% and a specificity of 62% at the cutoff value of 2.25 (10^−2^ AU). For predicting the difference between NPDR and PDR ✓SAF: AUC was 0.75 with a sensitivity of 81% and a specificity of 62% at the cutoff value of 2.32 (10^−2^ AU)	(47)

T2DM, type 2 diabetes mellitus; DR, diabetic retinopathy; NPDR, non-proliferative DR; PDR, proliferative DR; NCs, nailfold capillaries; NFC, nailfold capillaroscopy; AUC, area under the curve; SAF, skin autofluorescence; T1DM, type 1 diabetes mellitus; AGEs, advanced glycation end-products.

This review provided researchers with vast and absolute knowledge about the current schemes and drawbacks in non-ocular and non-invasive diagnostic biomarkers of DR. Although these non-invasive diagnostic biomarkers have only moderate accuracy, we still consider them to be of diagnostic value and sufficient accuracy for their use by non-ophthalmologists in primary screenings for DR, particularly in the absence of fundus investigations. Moreover, combinatorial biomarkers merit consideration since they have substantially greater sensitivity compared to individual biomarkers. Consequently, extensive investigations and validations are necessary to determine whether specific non-ocular biomarkers or their combinations exhibit the greatest predictive efficacy for use as a screening tool in routine clinical practice for non-ophthalmologists.
